# Adaptive Cellular Responses following SARS-CoV-2 Vaccination in Primary Antibody Deficiency Patients

**DOI:** 10.3390/pathogens13060514

**Published:** 2024-06-18

**Authors:** Sudhir Gupta, Houfen Su, Sudhanshu Agrawal, Yesim Demirdag, Michelle Tran, Sastry Gollapudi

**Affiliations:** Program in Primary Immunodeficiencies, Division of Basic and Clinical Immunology, University of California at Irvine, Irvine, CA 92697, USA; hsu@hs.uci.edu (H.S.); sagrawal@uci.edu (S.A.); yyilmazd@hs.uci.edu (Y.D.); thumt@hs.uci.edu (M.T.); svgollap@uci.edu (S.G.)

**Keywords:** COVID-19 vaccine, CVID, CD4 Treg, CD8 Treg, TFR, Breg, Cytotoxic T cells, IFNγ

## Abstract

Since the start of the COVID-19 pandemic, in a short span of 3 years, vaccination against SARS-CoV-2 has resulted in the end of the pandemic. Patients with inborn errors of immunity (IEI) are at an increased risk for SARS-CoV-2 infection; however, serious illnesses and mortality, especially in primary antibody deficiencies (PADs), have been lower than expected and lower than other high-risk groups. This suggests that PAD patients may mount a reasonable effective response to the SARS-CoV-2 vaccine. Several studies have been published regarding antibody responses, with contradictory reports. The current study is, perhaps, the most comprehensive study of phenotypically defined various lymphocyte populations in PAD patients following the SARS-CoV-2 vaccine. In this study, we examined, following two vaccinations and, in a few cases, prior to and following the 1st and 2nd vaccinations, subsets of CD4 and CD8 T cells (Naïve, T_CM_, T_EM_, T_EMRA_), T follicular helper cells (T_FH1_, T_FH2_, T_FH17_, T_FH1/17_), B cells (naïve, transitional, marginal zone, germinal center, IgM memory, switched memory, plasmablasts, CD21^low^), regulatory lymphocytes (CD4Treg, CD8Treg, T_FR_, Breg), and SARS-CoV-2-specific activation of CD4 T cells and CD8 T cells (CD69, CD137), SARS-CoV-2 tetramer-positive CD8 T cells, and CD8 CTL. Our data show significant alterations in various B cell subsets including Breg, whereas only a few subsets of various T cells revealed alterations. These data suggest that large proportions of PAD patients may mount significant responses to the vaccine.

## 1. Introduction

Severe acute respiratory syndrome coronavirus 2 (SARS-CoV-2) emerged late in 2019 and caused the coronavirus disease 2019 (COVID-19) pandemic. Vaccines were developed quickly, became available in the end of 2020, and had a tremendous impact on protection from SARS-CoV-2 mortality. In the last four years, the SARS-CoV-2 vaccine has changed the landscape of the COVID-19 pandemic. On 5 May 2023, more than three years since COVID-19 was designated as a pandemic, the World Health Organization (WHO) declared an end to the pandemic. As of 4 February 2024, over 774 million confirmed cases and more than seven million deaths have been reported globally. Globally, the number of new cases decreased by 58% during the 28-day period from 8 January to 4 February 2024 as compared to the previous 28-day period, with over 503,000 new cases reported. The number of new deaths decreased by 31% as compared to the previous 28-day period, with over 10,000 new fatalities reported. During the period from 8 January to 4 February 2024, both new COVID-19 hospitalizations and admissions to an intensive care unit (ICU) recorded an overall decrease of 32% and 38%, respectively.(WHO monthly report). The two most commonly used mRNA vaccines in the US are 94–95% effective in reducing the severity of COVID-19 [1,2,3]. Immune responses in healthy subjects following SARS-CoV-2 infections and to SARS-CoV-2 vaccines have been extensively studied [4,5,6,7,8,9,10,11,12,13,14,15,16,17]. Patients with IEI, especially primary antibody deficiency diseases (PADs), are at an increased risk for contracting SARS-CoV-2 infection [18]; however, many of these patients either did not have severe disease or recovered from severe disease [19,20,21,22,23,24,25,26,27]. Several studies have reported on the immune responses to infections with SARS-CoV-2 in patients with primary antibody deficiency [28,29,30]. The response to the vaccine in primary antibody deficiencies (PADs), in most studies, assessed the humoral and cellular immunity following a two- or three-dose mRNA vaccination. In these studies, humoral immunity was evaluated using many different quantitative and qualitative methods, such as measuring specific antibody levels against full spike protein (S) or receptor binding domain (RBD) or various neutralization assays. SARS-CoV-2 cellular immunity was evaluated using IFNγ release assays (IGRA), proliferation assays, cytokine release assays, or measurement of activation markers by flow cytometry.

The majority of these studies have reported that patients with PADs generated specific antibodies following the second and third doses of the SARS-CoV-2 vaccine [31,32,33,34,35,36,37]. Some studies showed a normal or almost normal T cell response to the vaccine [36,37], whereas other studies demonstrated a lower response in patients with IEI when compared to healthy controls [32,38,39]. However, none of these studies reported a detailed analysis of lymphocyte subsets.

Here, we report, perhaps, the most comprehensive analysis of subsets of CD4+, CD8+, T follicular helper cells (T_FH_), B cells, and four different members of the regulatory lymphocyte club in patients with PADs. Our data show major abnormalities in subsets of B cells.

## 2. Materials and Methods

### 2.1. Subjects

Fifty-four healthy controls (HC) and 42 patients with PADs were studied. The demographic data are shown in Table 1.

Among the 42 PAD patients, 30 were diagnosed with CVID; the diagnosis was made according to the ESID and Pan American criteria [40]. The patients with hypogammaglobulinemia had low total IgG, normal IgA and IgM, and an impaired response to the Penumovax-23 vaccine. Secondary causes of hypogammaglobulinemia were excluded. The patients with sIgMD, IgG subclass deficiency, XLA, and SAD had an impaired response to Penumovax-23. SAD was characterized by normal IgG, IgA, IgM, and IgG subclasses and an impaired response to Pneumovax-23. Therefore, all patients in our cohort had an impaired response to the T-independent lipopolysaccharide vaccine. The age in the HC ranged from 23 years to 72 years and, in the PAD group, ranged from 17 years to 80 years. Sixteen males and 38 females were in the HC group, whereas in the PAD group, there were 20 males and 22 females. Fourteen HC received the Moderna vaccine, and 40 HC received the Pfizer vaccine, whereas 9 received the Moderna vaccine, and 33 received the Pfizer vaccine in the PAD group. Only one patient with specific antibody deficiency (SAD) was exposed to SARS-CoV-2 virus infection. All patients were receiving immunoglobulin replacement therapy (IgRT). All patients and HC had received two doses of the mRNA SARS-Co-V2 vaccine (Pfizer or Moderna), and blood was drawn within 1–2 months of the second dose of the vaccine and in patients prior to the next dose of immunoglobulin infusion. In three CVID patients and 2 HC, studies were also performed longitudinally before and following the 1st and 2nd doses of the Pfizer vaccine.

### 2.2. Antibodies and Reagents

The following monoclonal antibodies and their isotype controls were purchased from various sources: BioLegend (San Diego, CA, USA)—CXCR5 AL488, CCR6 BV650, CD38 BV 650, CD127 BV510, CD127 PE, CD4 BV650, CD4 Percp, CD8 Percp, CD8a BV605, CD8BV421, CD8a AL700, CD24 BV510, CCR7 BV510, CD45RA BV650, CCR6 BV650, CD45RA BV510, CD45RA BV650, CXCR3 BV421, CD19 Percp, CD20 Percp, CD69BV510, CD69 AL700, CD107a PE, Granzyme B AL647, Perforin FITC; BD Biosciences (San Jose, CA, USA)—CD25 APC, FoxP3 PE, Mouse IgG1PE, CD25 FITC, ICOS AL647, PD-1 PE, CD27 FITC, IgD PE, IgM APC, CD21 BV421,CD86 PE IFN gamma FITC, CD183 PE, CD38 APC, CD38 FITC.

MLB International (Woburn, MA, USA)—HLA-A*02:01 SARS-CoV-2 Spike Glycoprotein Tetramer YLQPRTFLL. SARS-CoV-2 (2019-nCoV) Spike S1+S2 ECD-His Recombinant Protein (Protein Construction: A DNA sequence encoding the SARS-CoV-2 (2019-nCoV) Spike Protein (S1+S2 ECD) (YP_009724390.1) (Val 16-Pro1213) expressed with a polyhistidine tag at the C-terminus) was purchased from Sino Biological, Houston, Texas; FOX P3 Buffer set, Cytofix/cyotoperm plus kit, and fixation permeabilization kit with Golgistop were purchased from BD Biosciences (San Diego, CA, USA).

### 2.3. Immunophenotyping

Approximately 1 million peripheral blood mononuclear cells (PBMC) were used per combination for antibody staining. A total of 20 μL of antibody was added to the PBMC for 30 min. The PBMC were washed and fixed with 2% paraformaldehyde (PFA).

All fluorescence minus one (FMO) controls and isotype controls were stained and fixed with 2% PFA for flow cytometry. The cells were acquired with a BD FACS Celesta (Becton-Dickenson, San Jose, CA, USA) equipped with a BVR laser. Forward and side scatter and singlets were used to gate and exclude cellular debris. Thirty thousand cells were acquired and analyzed using FlowJo software version 10.10.0 (Ashland, OR, USA).

B cell and B cell subsets were identified by the following cell surface markers: naïve B cells—CD20+CD27−IgD+IgM+, transitional B cells—CD20+CD38+IgM++, marginal zone (MZ) B cells—CD20+CD27+IgD+IgM+, IgM memory B cells—CD20+/CD27+IgM+, GC B cells—CD20+IgD-CD27+CD38+, class switch memory (CSM) B cells—CD20+CD27+IgD-IgM-, plasmablasts—CD20+CD38++IgM−, mature B cells—CD21^high^CD20+CD38−, CD21^Low^ cells—CD20+CD38-CD21^low^.

The following cell surface phenotypes were used to identify subsets of CD4 T cells and CD8+ T cells: naïve (T_N_)—CD4+/CD8+CD45RA+CCR7+, central memory (T_CM_)—CD4+/CD8+CD45RA−CCR7+, effector memory (T_EM_)—CD4+/CD8+CD45RA−CCR7−, CD45RA+ effector memory, terminally differentiated effector memory (T_EMRA_)— CD4+/CD8+CD45RA+CCR7−.

T follicular helper cells were identified with the following markers: cT_FH_—CD4+/CXCR5+CD45RA−, T_FH_1—CD4+CXCR5+CD45RA−CCR6−CXCR3+, T_FH_2—CD4+CXCR5+CD45RA−CCR6−CXCR3−, T_FH_17—CD4+CXCR5+CD45RA−CCR6+CXCR3, T_FH_1+T_FH_17—CD4+/CXCR5+/CD45RA−/CCR6+/CXCR3+.

For regulatory cells, the cells after surface staining were fixed and permeabilized with a Foxp3 staining buffer set (BD Bioscience) as per the manufactures protocol and intracellularly stained with anti-Foxp3PE monoclonal antibody, and an appropriate isotype control (Mouse IgG 1,k-PE) was used to evaluate nonspecific staining.

Regulatory lymphocytes were identified with the following markers: CD8Treg—CD8+CD183+CCR7+CD45RA−, CD4Treg—CD4+CD25+CD127− Foxp3+, T_FR_—CD4+CCR5+CD45RA−CD25^high^ FoxP3+, and Breg—CD19+CD24+CD38+.

For SARS-CoV-2-specific T cells, tetramer-positive T cells were analyzed by the following technique. A total of 200 μL blood was mixed with 5 μL CD8PerCP monoclonal antibody and 10 μL HLA-A*0201 spike Tetramer PE (HLA-A*02:01 SARS-CoV-2 Spike Glycoprotein Tetramer YLQPRTFLL), vortexed gently, and incubated for 30 min at room temperature protected from light. Red blood cells were lysed using 1 mL of lyse reagent supplemented with 0.2% formaldehyde fixative reagent per tube. The tubes were centrifuged at 150× *g* for 5 min, and the supernatants were removed. Three milliliters of FACS buffer was added, and the tubes were centrifuged at 150× *g* for another 5 min. The cell pellets were resuspended in 500 μL of phosphate-buffered saline (PBS) and 0.1% formaldehyde and stored at 4 °C for 1 h in the dark prior to analysis by flow cytometry.

Functional cytotoxic CD8 T cells (CTLs) were analyzed by the following technique: A total of 200 µL blood sample was incubated for 30 min with CD8PerCP, fixed and permeabilized by Fix Perm buffer (BD biosciences, San Diego, CA, USA), and then incubated with granzyme B AL647 and Perforin FITC monoclonal antibodies (MLB International, Woburn, MA, USA) and an appropriate isotype control.

### 2.4. In Vitro Activation by SARS-CoV-2 Spike Protein

Peripheral blood mononuclear cells (PBMC; 4 × 10^6^/mL) were activated with SARS-CoV-2 spike protein (2 µg/mL) at 37 °C. Four microliters of BD Golgi Stop for every 6 mL of cell culture was added and incubated for an additional 5 h. The cells were stained with Percp-labeled anti-CD4, PE-labeled anti-CD8, Brilliant Violet 510-labeled anti-CD69, and Brilliant Violet 421-labeled anti-CD137, according to the manufacturer’s protocol. For the detection of activation markers on the B lymphocytes, the cells were stained with Percp labeled anti-CD20, BV510 labeled CD69, and PE-labeled anti-CD86. The cells were then washed and fixed with Cytofix/Cytoperm TM (BD Biosciences, San Diego, California) and stained with FITC-labeled anti-IFN-γ or appropriate isotypic control (Mouse IgG 1, k-FITC). Negative controls with no peptide stimulation were run in parallel for each sample. All samples were acquired on a BD FACS Celesta (BD Biosciences) flow cytometer and analyzed using FlowJo Software. Background expression of CD69 and CD86 and intracellular IFNγ in the negative control were subtracted from those in the antigen-stimulated samples for each response prior to further analysis.

**Figure 1:**
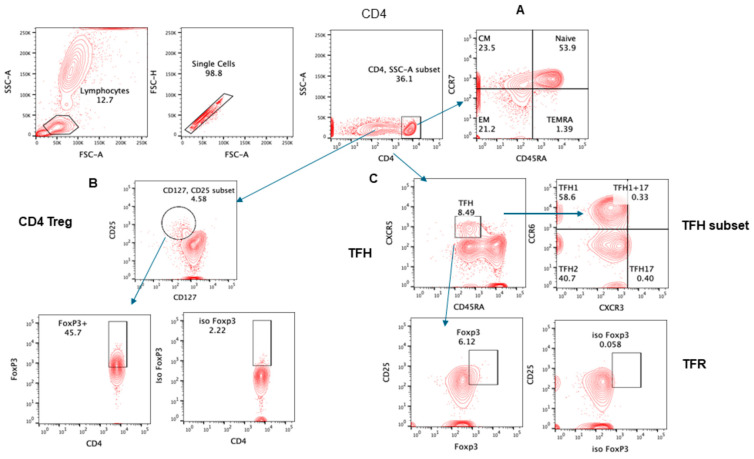
Gating strategy for subsets of CD4+ T cells. Contour plot was used for gating strategy; gated lymphocytes were analyzed for singlet and CD4-expressing cells. These CD4+ cells were further analyzed for (**A**) CD4 subsets naïve: CCR7+CD45RA+, central memory (CM): CCR7+CD45RA−, effector memory (EM): CCR7−CD45RA−, and T effector memory: CD45RA+ (T_EMRA_) CCR7− CD45RA+. (**B**) Treg CD25+CD127 ^low^ cells expressing FoxP3. (**C**) Follicular helper cells (T_FH_) CXCR5+CD45RA−; subsets of T_FH_ cells were further analyzed—T_FH1_: CXCR3+CCR6−, T_FH2_: CXCR3−CCR6−, T_FH1_7: CXCR3−CCR6+.

**Figure 2:**
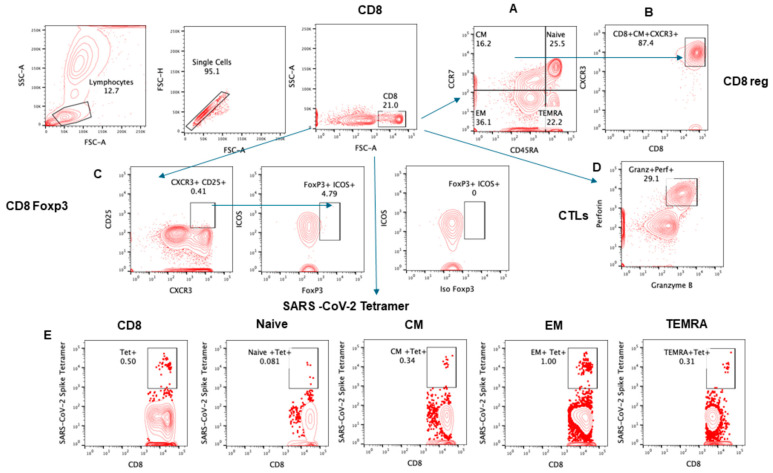
Gating strategy for subsets of CD8+ T cells. Contour plot was used for gating strategy; gated lymphocytes were analyzed for singlet and CD8-expressing cells. These CD8+ cells were further analyzed for (**A**) CD4 subsets naïve: CCR7+CD45RA+, central memory (CM): CCR7+CD45RA−, effector memory (EM): CCR7−CD45RA−, and T effector memory RA+ (TEMRA): CCR7−CD45RA+. (**B**) CD8Treg CXCR3 expressing CM: CCR7+CD45RA−. (**C**) FoxP3 and ICOS+ expression in CD25+CXCR3+ CD8 cells. (**D**) CD8+ cells were analyzed for granzyme B and perforin expression. (**E**) SARS-CoV-2 spike protein-specific, tetramer-positive cells in total CD8 and subsets.

**Figure 3:**
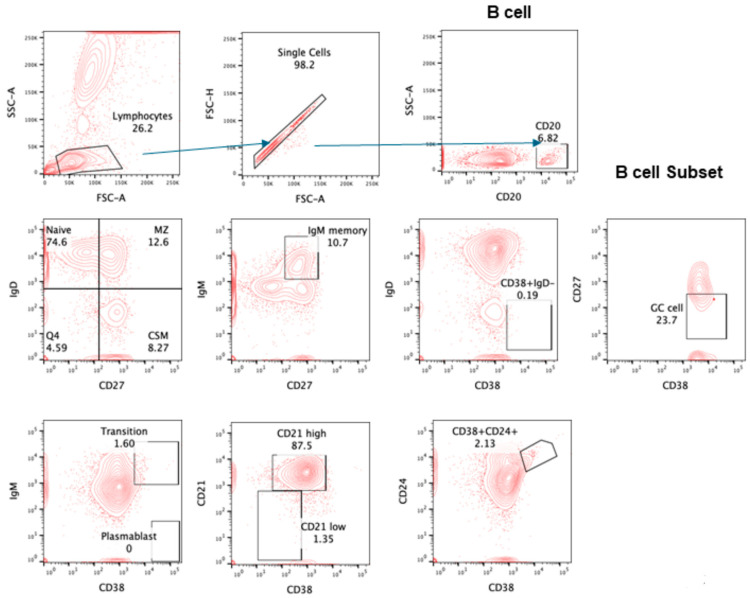
Gating strategy for subsets of CD20+ B cells. Contour plot was used for gating strategy; gated lymphocytes were analyzed for singlet and CD20-expressing B cells. These B cells were further analyzed for naïve: IgD+CD27−, MZ: IgD+CD27+, CSM: IgD−CD27+, IgM memory: IgM+CD27+, transition B cells: IgM+CD38+, plasma blast: IgM−CD38+, CD21^High^ and CD21^Low^, Breg: CD24+CD38+, and GC cells: IgD−CD38+CD27−.

**Figure 4:**
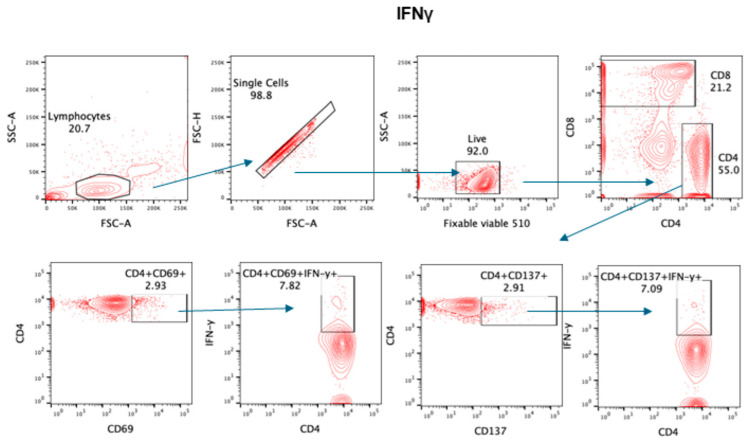
Gating for intracellular IFNγ. Representative contour plot was used for gating strategy; gated lymphocytes were analyzed for singlet, live, and CD4/CD8-expressing cells. These CD4+ cells were further analyzed for IFNγ in CD69+ and CD137+ cells.

The gating strategy for various lymphocyte populations and subpopulation, CTLs, and intracellular IFNγ are shown in Figure 1, Figure 2, Figure 3 and Figure 4.

### 2.5. Statistical Analysis

GraphPad Prism version 10.2.1 (GraphPad Inc., San Diego, CA, USA) was used. Unpaired *t*-test was used for comparisons between two groups. *p* value of <0.05 was considered statistically significant.

## 3. Results

### 3.1. Immune Responses following Two Doses of SARS-CoV-2 mRNA Vaccine in PAD Patients

#### 3.1.1. Subsets of CD4 and CD8 T Cells in PAD following Two Vaccinations by mRNA Vaccine

**Figure 5:**
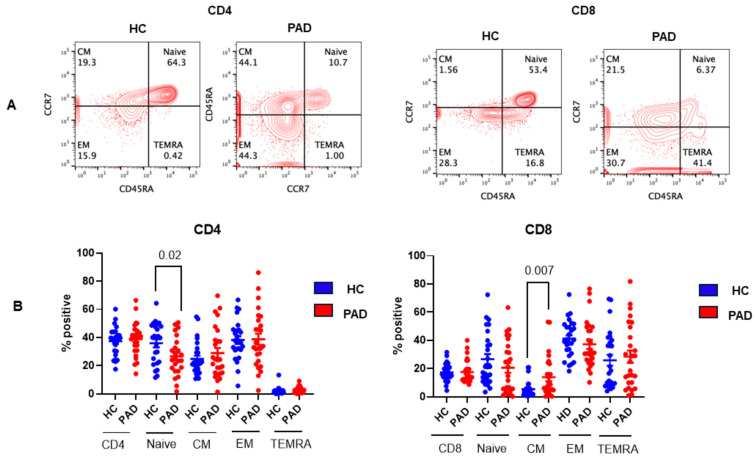
Subsets of CD4+ and CD8+ T cells in the HC and PAD patients following two doses of SARS-CoV-2 vaccine. A representative contour flow cytograph is shown (**A**). Cumulative data show significantly decreased naïve CD4+ T cells and significantly decreased CD8T_CM_ cells in PAD patients as compared to the HC (**B**).

Naïve CD4 and CD8 T cells, upon activation by an antigen, undergo activation, proliferation, and generation of effector and memory T cells [41,42,43]. Memory T cells that migrate to the lymph nodes are termed central memory (T_CM_), and those that migrate to extra-lymphoid tissue are termed effector memory (T_EM_). T effector memory cells that re-acquire CD45 are termed T_EMRA_. These subsets are distinct in their phenotype and functions [44]. In this study, we studied these subsets. A representative flow cytograph in the HC and PAD patients is shown in Figure 5A, and cumulative data on PAD patients are shown in Figure 5B. Naïve CD4+ T cells were significantly decreased (*p* < 0.03), whereas CD8 T_CM_ cells were significantly increased (<0.007) in the PAD group as compared to the HC.

#### 3.1.2. SARS-CoV-2 Specific T Cells following Two Doses of SARS-CoV-2 Vaccine

**Figure 6:**
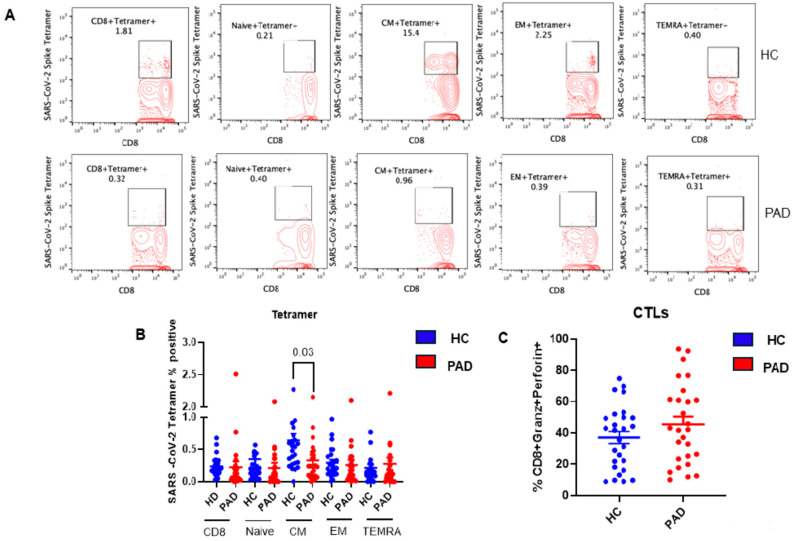
SARS-CoV-2-specific, tetramer-positive CD8+ T cell subsets and CD8 CLT cells following two doses of SARS-CoV-2 vaccine. A representative contour flow cytograph for IFNγ+ in various subsets of CD8+ T cells in the HC and PAD patients (**A**). Cumulative data for the HC and PAD patients for IFNγ+ CD8 T cell subsets (**B**) and CD8 CTL cells (**C**) show significantly decreased tetramer-positive CD8 T_CM_ cells in the PAD patients (**B**).

At the baseline (without in vitro activation with spike protein), tetramer positive for SARS-CoV-2 CD8+ T cells were studied in all four subsets. In addition, CTLs were studied in CD8+ T cells positive for granzyme and perforin. A representative flow cytograph is shown in Figure 6A, and cumulative data are shown in Figure 6B. Tetramer-positive CD8 T_CM_ cells were significantly decreased (*p* < 0.022) in the PAD patients as compared to the HC; however, CTLs were comparable in the two groups.

**Figure 7:**
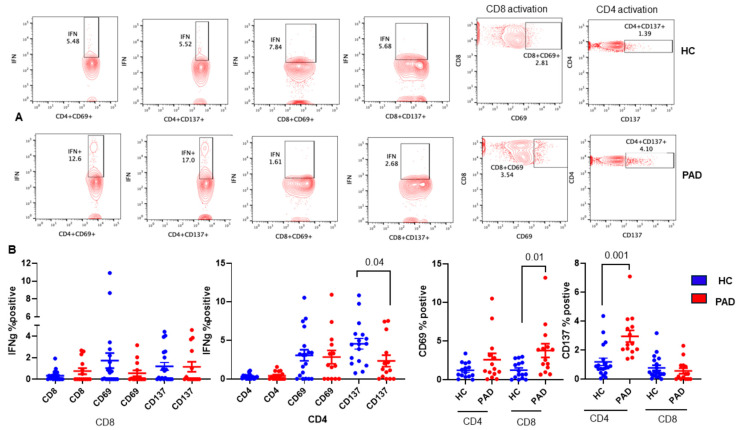
Effect of in vitro activation with SARS-CoV-2 protein on activation of CD4 and CD8 T cells and IFNγ+ CD4 and CD8 T cells. PBMC were activated with SARS-CoV-2 spike protein, activation of CD4 and CD8 was examined by the expression of CD69 and CD137, and intracellular IFNγ was examined in CD69+ and CD137+ CD4 and CD8 T cells. A representative contour flow cytograph for HC and PAD is shown (**A**). Cumulative data show significantly increased CD8+CD69+ T cells and CD4+CD137+ T cells and significantly decreased C4+CD137+IFNγ+ T cells in the PAD group (**B**).

We also examine SARS-CoV-2-specific T cell responses by stimulating PBMC with SARS-CoV-2 protein, and expression of CD69 and CD137 as activation markers was studied in both CD4 and CD8+ T cells. Intracellular IFNγ was examined in CD69+ and CD137+ subsets of both CD4 and CD8+ T cells. A representative of activation markers CD69 and CD137 is shown in Figure 7A, and cumulative data for both activation markers and intracellular IFN-γ in CD69+ and CD137+, CD4+, and CD8+ T cells are shown in Figure 7B. CD8+CD69+ (*p* < 0.009) and CD4+CD137+ (<0.001) subsets were significantly increased in the PAD group as compared to the HC. CD4+CD137+IFNγ+ cells were significantly decreased (*p* < 0.037) in the PAD group as compared to the HC.

#### 3.1.3. T_FH_ Cells following Two Doses of mRNA SARS-CoV-2 mRNA Vaccine

**Figure 8:**
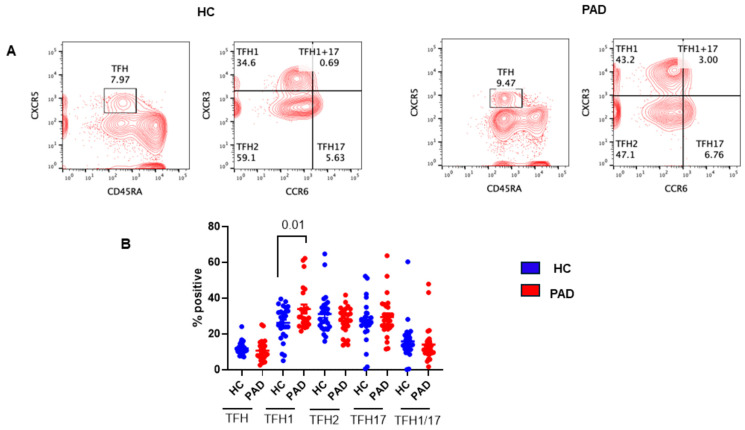
T_FH_ cells and subsets of T_FH_ cells in HC and PAD following two doses of SARS-CoV-2 vaccine. A representative contour flow cytograph in HC and PAD (**A**). Cumulative data show a significant increase in T_FH1_ cells (**B**).

The T_FH_ cells are major CD4+ T helper subsets that are essential for B cell differentiation into immunoglobulin-producing plasma cells as well as for germinal center (GC) formation and generation of memory B cells [45,46,47]. The GC is the primary site for class-switched DNA recombination and affinity maturation. T_FH_ cells in the GC regulate class-switched DNA recombination and selection of high-affinity, antibody-producing B cells. According to the expression of CXCR3 and CCR6 on CD4+CXCR5+ T_FH_ cells, they are divided into three different subsets of T_FH_ cells with different functions [48]. They include T_FH_1, T_FH_2, T_FH_1/T_FH_17, and T_FH_17; all are able to efficiently induce an antibody response by memory B cells. Therefore, we studied all subsets of T_FH_ in the PAD patients. A representative flow cytograph is shown in Figure 8A, and cumulative data are shown in Figure 8B. T_FH1_ cells were significantly increased (*p* < 0.012) in the PAD group as compared to the HC.

#### 3.1.4. B Cell Subpopulations following TWO Doses of SARS-CoV-2 mRNA Vaccine

**Figure 9:**
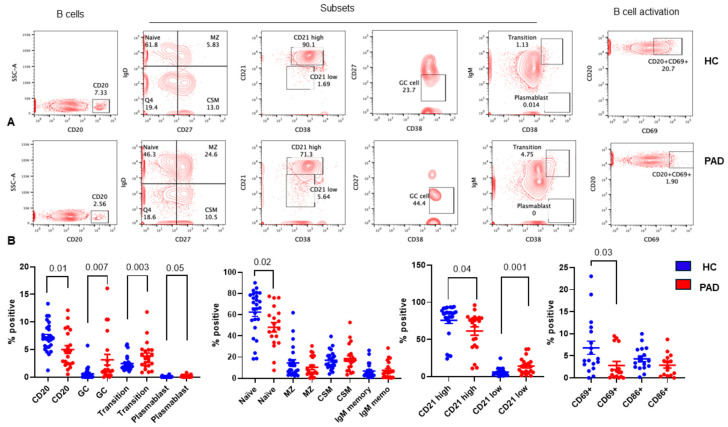
B cells and B cell subsets in HC and PAD following two doses of SARS-CoV-2 vaccine. Various subsets of B cells and activation by the expression of CD69 and CD86 on CD20+ B cells were examined. A representative contour flow cytograph is shown in (**A**). Cumulative data (**B**) show significantly reduced naïve B cells and CD20+ B cells and significantly increased transitional B cells, GC B cells, and plasmablasts in the PAD group as compared to the HC.

B cells, after being released from the bone marrow, migrate to the spleen and lymphocytes to undergo activation and differentiation into short-lived plasmablasts and long-lived plasma cells and generation of memory B cells [49,50,51,52,53]. Transitional B cells mature into naïve B cells that migrate to the marginal zone (MZ) or GC. MZ B cells undergo activation and differentiation to short-lived plasmablasts and IgM memory B cells. In the GC, B cells undergo isotype class-switching and somatic hypermutation and differentiation to long-lived plasma cells and class-switched memory B cells. We studied all subsets of B cells following two doses of the SARS-CoV-2 vaccine in the PAD group and the HC. A representative flow cytograph is shown in Figure 9A, and cumulative data are shown in Figure 9B. CD20+ B cells (*p* < 0.029) and naïve B cells (*p* < 0.04) were significantly decreased, whereas transitional B cells (*p* < 0.002), GC B cells (*p* < 0.005), plasmablasts (*p* < 0.046), and CD21^low^ B cells (*p* < 0.001) were significantly increased in the PAD group compared to the HC. B cell activation was examined by the expression of CD69 and CD86 on CD20+ B cells. CD69-expressing B cells were significantly decreased (*p* < 0.03) in the PAD group as compared to the HC.

#### 3.1.5. Regulatory Lymphocytes following Two Doses of SARS-CoV-2 Vaccine

**Figure 10:**
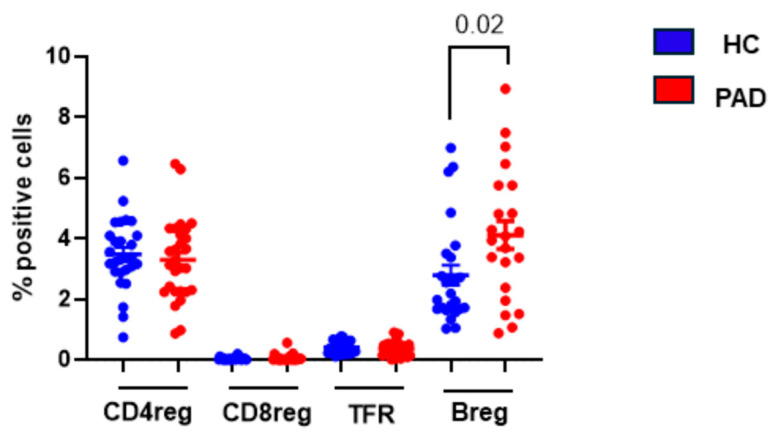
Regulatory lymphocytes following two doses of SARS-CoV-2 vaccine. CD4Treg, CD8Treg, TFR, and Breg were examined in the HC and CVID patients. Data show significantly increased Breg cells in CVID patients as compared to the HC.

Peripheral tolerance is induced by CD4Treg, CD8Treg, T_FR_, and Breg cells that regulate GC reaction by multiple mechanisms, including anergy, apoptosis, and suppression of effector functions of self-reacting T and B cells [reviewed in [54]]. Since patients with PAD, especially CVID, develop autoimmunity and their alterations have been reported in CVID [55], we studied their relative proportions following two doses of the SARS-CoV-2 vaccine. The data are shown in Figure 10. Breg cells were significantly increased (*p* < 0.02) in the PAD group as compared to the HC. No significant difference was observed in any other regulatory lymphocytes between the PAD group and the HC.

### 3.2. Immune Responses before and following 1st and 2nd Doses of SARS-CoV-2 Vaccine

#### 3.2.1. Subsets of CD4 and CD8 T Cells before and following 1st and 2nd Doses of SARS-CoV-2 Vaccine

**Figure 11:**
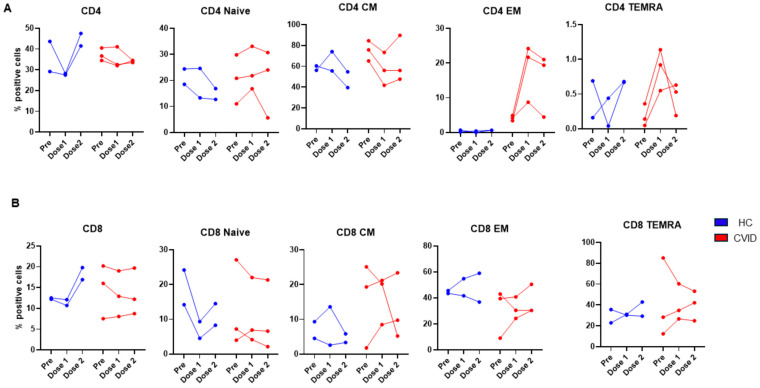
Subsets of CD4 (**A**) and CD8 (**B**) prior to and following 1st dose and 2nd doses of SARS-CoV-2 vaccine. Though variable changes were observed in both HC and CVID, CVID patients exhibited increased CD4_TEM_ and CD4_TEMRA_ as compared to HC (**A**). CD8 responses were highly variable in both HC and CVID.

Two HC and three patients with CVID were studied before and following the 1st dose (prior to the 2nd dose) and after the 2nd dose (1 month after) of the SARS-CoV-2 vaccine (Pfizer). None of the HC and only one CVID patient had a prior history of exposure to SARS-CoV-2. The data for CD4+ T cell subsets are shown in Figure 11A. CD4+ T cells in the HC were increased following the 2nd dose, whereas in the patients, no significant change was observed. CD4 naïve T cells decreased in the HC, whereas in the patients, the changes were variable, ranging from no change to a modest increase or decrease following the 2nd dose. CD4 T_CM_ decreased in the HC after the 2nd dose, whereas in the CVID patients, CD4 T_CM_ cells decreased following the 1st dose and modestly recovered following the 2nd dose. CD4 T_EM_ cells in the HC were very low and did not change, whereas in the CVID patients, CD4 T_EM_ increased markedly following the 1st dose and remained high in two of the three subjects. In the patients, CD4_TEMRA_ markedly increased following the 1st dose and remained high following the dose in two of the three patients.

The data on CD8+ T cell subsets are shown in Figure 11B. Following the 2nd dose of the vaccine, CD8+ T cells increased in the HC; however, they did not change in the CVID patients. In the HC, naive CD8 T cells decreased following the 1st dose and modestly recovered following the 2nd dose, whereas in two of the three patients, naïve CD8 T cells decreased following the 1st dose and did not recover following the 2nd dose. In two of the three patients, CD8 T_CM_, CD8_TEM_, and CD8_TEMRA_ cells increased following the 1st dose and increased further after the 2nd dose, whereas variable changes were observed following the 2nd dose in the HC.

#### 3.2.2. Subsets of T_FH_ cells before and following 1st and 2nd doses of SARS-CoV-2 vaccine

**Figure 12:**
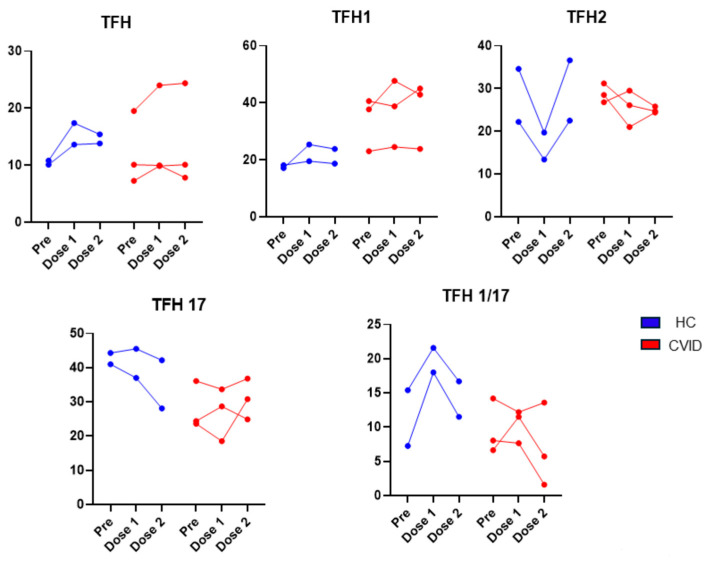
Subsets of T_FH_ cells prior to and following 1st dose and 2nd doses of SARS-CoV-2 vaccine. Basal levels of T_FH1_ were higher and remained high after vaccination in CVID. TFH2 were markedly reduced after the 1st dose in HC, whereas they were modestly reduced in CVID. T_FH1/17_ increased after the 1st dose in HC, whereas in CVID, two of three subjects showed no increase.

The effect of vaccination in T_FH_ cells is shown in Figure 12. T_FH_ cells increased in the HC following the 1st dose, and no further increase was observed following the 2nd dose. Two of the three CVID patients had no change in T_FH_ cells. No changes were observed in T_FH1_ in the HC and in two of the three CVID patients following the 1st vaccination. T_FH2_ cells decreased in the HC following the 1st dose but recovered following the 2nd dose of the vaccine. Two of the three CVID patients also demonstrated a decrease in T_FH2_ cells following the 1st dose of the vaccine; however, they remained low following the 2nd dose of the vaccine. T_FH17_ cells decreased following the 2nd vaccination in the HC; however, they did not change much in two of the three patients. T_FH1/17_ cells markedly increased following the 1st vaccination and decreased following the 2nd dose but remained higher than pre-vaccination.

#### 3.2.3. Subsets of B Cells and Regulatory Lymphocytes before and following 1st and 2nd Doses of SARS-CoV-2 Vaccine

**Figure 13:**
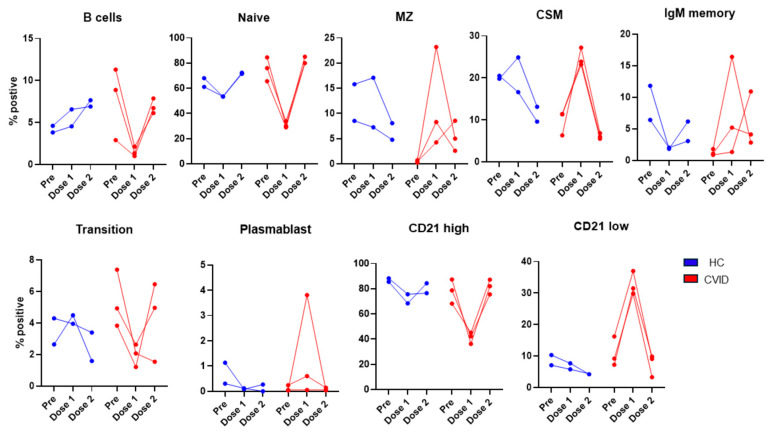
Subsets of B cells prior to and following 1st dose and 2nd dose of SARS-CoV-2 vaccine. CD20+ B cells increased after 1st and 2nd doses of vaccine, whereas CVID patients showed a marked decrease following 1st dose and recovered, to a large extent, following 2nd dose. Following 1st dose of vaccine, MZ, CSM, IgM memory, plasmablasts, and CD21low increased markedly, and transitional cells decreased markedly in CVID as compared to HC.

The data on B cell subsets are shown in Figure 13. B cells in the HC increased following the 1st and 2nd doses of the vaccine, whereas in the patients, B cells decreased following the 1st dose and returned towards baseline levels following the 2nd dose of the vaccine. Naïve B cells decreased following the 1st dose and returned to pre-vaccination levels following the 2nd dose of the vaccine in both the HC and patients. Transitional B cells decreased following the 1st dose and recovered following the 2nd dose of the vaccine. Transitional B cells decreased after the 2nd dose, and MZ B cells decreased in the HC, whereas in the patients, MZ B cells increased following the 1st dose of the vaccine and decreased following the 2nd dose but remained higher than pre-vaccination. CSM B cells in the HC decreased following the 2nd dose. In the patients, CSM markedly increased following the 1st dose and returned to the pre-vaccination level following the 2nd dose. In the patients, plasmablasts increased after the 1st dose and returned to the original levels after the 2nd dose. In CVID, plasmablasts were at the pre-vaccination levels following the 2nd dose of the vaccine. CD21^low^ B cells markedly increased following the 1st dose of the vaccine and returned to the pre-vaccination level. In contrast, in the HC, CD21^low^ cells decreased after the 1st dose and further decreased after the 2nd dose of the vaccine.

#### 3.2.4. Regulatory Lymphocytes before and following 1st and 2nd Doses of SARS-CoV-2 Vaccine

**Figure 14:**
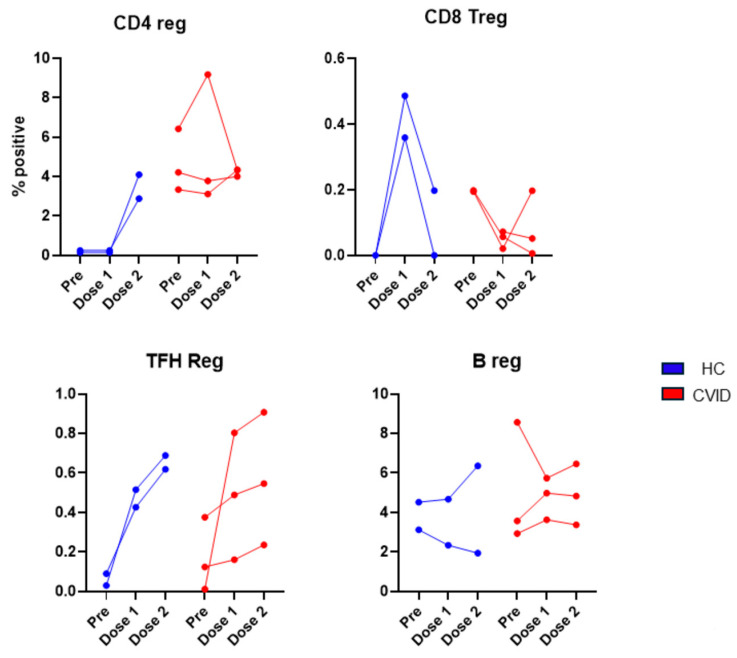
Regulatory lymphocytes prior to and following 1st dose and 2nd dose of SARS-CoV-2 vaccine. CD4Treg increased after 2nd dose in HC; however, no such increase was observed in CVID. TFR cells increased after 1st and 2nd doses in both HC and CVID. A reverse effect was observed for CD8Treg. In HC, CD8Treg markedly increased whereas in CVID CD8Treg markedly decreased after 1st dose of vaccine.

The data of four members of the regulatory lymphocyte club (CD4Treg, CD8Treg, TFR, and Breg) are shown in Figure 14. CD4Treg cells were markedly increased in the HC following the 2nd dose of the vaccine, whereas in the CVID patients, no similar increase was observed. In the HC, CD8Treg cells were markedly increased following the 1st dose and returned towards the pre-vaccination levels. In the CVID patients, CD8Treg decreased following the 1st dose and did not recover completely. T_FR_ cells were increased in both the HC and patients. A modest increase in Breg was observed in two of the three patients following the 2nd vaccination.

#### 3.2.5. SARS-CoV-2-Specific, Tetramer-Positive and Cytotoxic T lymphocytes before and following 1st and 2nd Doses of SARS-CoV-2 Vaccine

**Figure 15:**
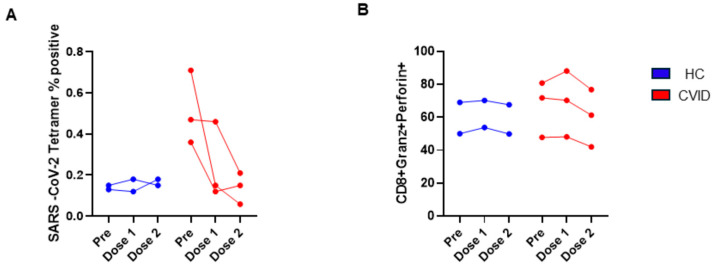
SARS-CoV-2 tetramer-positive (**A**) and CTL CD8 T cells (**B**) prior to and following 1st dose and 2nd dose of SARS-CoV-2 vaccine. SARS-CoV-2+ CD8+ T cells markedly decreased following 1st and 2nd vaccinations, and only minor changes were observed in HC (**A**). CTL CD8 T cells were similar in both groups.

Tetramer-positive CD8+ T cells did not change following the 1st or 2nd dose of the vaccine. Tetramer-positive CD8+ T cells were significantly higher in CVID prior to vaccination as compared to the HC; however, after the 2nd vaccination, tetramer-positive CD8+ T cells were markedly reduced and were comparable to the HC. No significant changes were observed in CTLs among both groups (Figure 15).

## 4. Discussion

In this study, we report, perhaps, the most comprehensive phenotypic analysis of various subpopulations of CD4+ T cells, CD8+ T cells, T_FH_ cells, B cells, and CD4Treg, CD8Treg, T_FR_, and Breg in PAD patients following two doses of the mRNA SARS-CoV-2 vaccine and changes in these subsets in a small number of CVID patients before and following the 1st dose and 2nd dose of the SARS-CoV-2 vaccination. Our data show major alterations in the subpopulation of B cells.

Several investigators have studied antibody responses to SARS-CoV-2 vaccination in PAD (especially in CVID). Most of these studies have been conducted after 2–4 doses of the vaccine, and both normal and impaired responses have been reported. Different reports have demonstrated rather good vaccination responses, with detectable humoral and cellular responses in up to 80% of CVID patients after the second vaccination [32,34,35,37].

Hagin et al. [35] reported that most patients with inborn errors of immunity (IEI) generate humoral and cellular immune responses to the Pfizer–BioNTech COVID-19 vaccine. Neutralizing anti-receptor-binding domain (RBD) antibodies, RBD-specific B cells of the IgG^+^ and IgA^+^ isotype, and T cells producing IL-2 and IFN-γ were detected in most vaccinated patients. They also reported the generation of memory B cells following two doses of the vaccine. They evaluated 26 patients with IEI, including eighteen with predominant antibody deficiencies and three with combined immunodeficiencies, and reported that, 2 weeks after the second dose of the Pfizer–BioNTech COVID-19 vaccine, 18 patients developed a specific antibody response, and 19 showed an S-peptide-specific T cell response. Gernez and colleagues [34] evaluated the immune response in 14 patients with predominant antibody deficiencies after the 3rd dose of the mRNA vaccination. They also observed that all 10 patients with CVID mounted an RBD IgG-specific antibody response, and all four specific antibody deficiency (SAD) or hypogammaglobulinemia patients mounted a positive RBD IgG-specific antibody response. These reports would be consistent with our results of increased plasmablasts in PADs following two doses of the vaccine.

Arroyo-Sanchez et al. [32] evaluated S1 antibody levels by ELISA in 18 patients with CVID and 50 healthy controls before and after the 2nd dose of the SARS-CoV-2 vaccines. After the second dose, 83% of CVID patients and 100% of HC became anti-S1 IgG positive. In another study, Murray et al. [36] reported that 70% of individuals with IEI and 64% of patients had detectable spike protein-specific antibody levels following the primary vaccination, whereas detectable specific antibody levels were found in 100% of healthy controls.

Milota et al. [56], in a prospective observational study, investigated humoral and cellular responses in 21 patients with CVID followed for 6 months. The study found that 11 of 21 (52.4%) patients with CVID and all individuals in the HC group had detectable anti-RBD SARS-CoV-2 antibodies 1 month after the 2nd dose of the BNT162b2 vaccine. No significant difference was observed in APRIL, BAFF, and IFN-α as potential humoral response markers that contribute to B cell maturation, survival, and class-switching between responders and non-responders. We also did not observe any significant difference in switched memory B cells between HD and PADs.

Ainsua-Enrich et al. [57] studied 22 SARS-CoV-2 uninfected patients with primary antibody deficiencies (PADs), including eleven patients with CVID and nine patients with uncharacterized PAD. They demonstrated positive seroconversion in 67% of the CVID patients 4 weeks after the 2nd dose of the vaccine, whereas all HC developed seroconversion.

Nielsen et al. [58] examined the spike protein RBD antibody (anti-S-RBD) levels after the second, third, and fourth doses of the mRNA SARS-CoV-2 vaccines in 33 patients with CVID. The third SARS-CoV-2 vaccination can increase the antibody levels in CVID patients. In addition, most of the two-dose non-responders seroconverted by repeating immunization.

Sauerwein et al. [38] examined isotype-specific and functional antibody responses six weeks after the second dose of the BNT162b2 vaccine in 31 adult patients with CVID as compared to 39 patients with milder forms of primary antibody deficiencies and 20 healthy controls. They observed that 48.4% of patients with CVID produced specific IgG levels comparable to HC and normal IgG responses. In contrast, CVID IgG non-responders showed defective vaccine-specific and superantigen-induced activation of both CD4+T cell subsets.

Sauerwin and colleagues [38] reported higher levels of MZ-like IgM memory B cells in CVID responder patients who responded to the SARS-CoV-2 peptide by making anti-SARS-CoV-2 antibodies. However, we did not observe any increase in MZ B cells or IgM memory B cells in our cohort. This discrepancy could be because we did not divide our patients into responders and non-responders. We have previously reported increased transitional B cells and IgM memory B cells and decreased GC and plasmablasts following SARS-CoV-2 infection in a patient with CVID [28]. In our current study, we observed a significant decrease in CD19+ B cells and an increase in transitional B cells, GC B cells, and plasmablasts in the PAD patients following two doses of the SARS-CoV-2 vaccine. A decrease in CD19+ cells in the PAD group as compared to the HC could be because, in the HC, CD19+ B cells increased following two doses of the vaccine, whereas in the PAD group, there might be an impaired response to the vaccine (Figure 13). In our longitudinal study, in the CVID patients, total B cells were decreased following the 1st dose and recovered after the 2nd dose of the vaccine that was shared by a decrease in naïve B cells and transition B cells and an increase in IgM memory and CSM B cells, plasmablasts, and CD21low B cells, suggesting a migration of B cells from the peripheral blood to lymphoid follicles and undergoing differentiation to memory and plasmablasts. However, the mechanism for their return to the baseline level remains unclear.

Fernandez Salinas and colleagues [33] reported decreased RBD antibodies and impaired memory B cells in CVID patients following two doses of the vaccine. Antoli et al. [59] evaluated 70 patients with primary and secondary immunodeficiencies, including 31 patients with CVID, and demonstrated an impaired antibody response (29%), low CD19+ peripheral B cells, and low switched memory B cells. However, we did not observe low switched memory B cells. It is interesting to note that, in CVID, switched memory B cells are decreased. Therefore, a comparable proportion of switched memory B cells in our cohort of PAD may suggest greater activation of switched B cells, which would be consistent with increased GC B cells and plasmablasts following two doses of the vaccine.

Another large, prospective, controlled, multicenter study looked at the humoral and cellular immune responses after two doses of the mRNA-1273 COVID019 vaccine in 505 patients with IEI and 192 controls [60]. The study measured anti-RBD antibodies, full spike (S) protein-specific binding, and neutralizing antibodies, and SARS-CoV-2-specific T cell responses were assessed by the IFN-γ release assay. After twenty-eight days following the second vaccine, seroconversion rates were 100% in HC, 81% in CVID, and 91% in combined immunodeficiency (CID), and neutralizing antibodies were detected in 100% of sera from HC and in patients with IgG deficiency/SAD, demonstrating that a majority of patients with antibody deficiency do respond to the SARS-CoV-2 vaccine. The levels of SARS-CoV-2-specific T cells using the IFN-γ release assay were significantly lower in the CVID cohort than in the controls (67% vs. 88%). In the present study, we also observed significantly lower (<0.037) SARS-CoV-2-specific CD4+CD137+IFN-γ+ cells.

CD21 forms a complex with CD19 and CD81 to act as a B cell co-receptor. There is a CD21−/^low^ memory B cell (MBC) population in PB that constitutes approximately 5% of the B cell pool, comprising both CD27+ and CD27− as well as switched and unswitched cells [61]. In humans, the generation/maintenance of CD21−/^low^ B cells is dependent on T cells, IL21R, and Tbet [62,63]. The CD21−/^low^ cells are antigen-experienced MBCs in a majority of conditions, as the cells are isotype-switched and express BCRs that have undergone somatic hypermutation. In critically ill COVID-19 patients, CD21−/^low^CD27− B cells have been observed to be expanded compared with healthy individuals [64]. Compared with vaccination-induced individuals, those induced by the infection showed better antigen-binding capacity and generated more CD21^low^ [64,65]. This population of B cell is distinct from other B cell subpopulations in that they resemble innate-like B cells and are increased in CVID [66,67]. In our current study, CD21^low^ B cells following two doses of the vaccine were also significantly increased (*p* < 0.001) in PAD patients as compared to the HC. CD21^low^ B cells proliferate poorly but make large amounts of antibodies. Therefore, their expansion would be consistent with increased plasma cells.

Follicular helper T (T_FH_) cells play a critical role in B cell differentiation into immunoglobulin-producing plasma cells and GC formation by their cognate receptor interactions and by their ability to produce IL-21 and IL-4 [68,69,70]. Besides T_FH_ cells, the GC formation is also regulated by follicular regulatory T (T_FR_) cells that express FoxP3 protein and can control the immunological synapse between T_FH_ and B cells [71,72].

According to the expression of CXCR3 and CCR6 markers, circulating T_FH_ (cT_FH_) cells are further classified as cT_FH1_ (CXCR5+CXCR3+CCR6−), cT_FH2_ (CXCR5+CXCR3−CCR6−), and cT_FH17_ (CXCR5+CXCR3−CCR6+) cells. All of them are able to induce in vitro antibody production by memory B cells, but only cT_FH2_ and cT_FH17_ are able to help naive B cells [48,73]. In the last decade, an understanding of the biology of T_FH_ and T_FR_ cells and their contribution to disease states has significantly increased [45]. Several studies have demonstrated a positive correlation between the proportion of different subtypes of cT_FH_ cells and the production of neutralizing IgG antibodies against certain viruses [74,75]. Additionally, disturbances in the cT_FH_ and cT_FR_ cell compartment are associated with the development and severity of autoimmune diseases [55,66].

Kasahara and colleagues [76] reported prior normal cT_FH_ cells but increased cT_FH17_ cells in patients with CVID who were not exposed to the SARS-Co-2 virus or vaccine and an increased ratio of cT_FH_/cT_FR_ cells in CVID patients with autoimmune diseases. However, cT_FH_ function in helping B cells to produce antibodies in vitro is preserved in CVID. In a COVID-19 convalescent CVID patient, we reported decreased T_FH_ and T_FH2_ cells and increased T_FH1_ and T_FH17_ cells, comparable to healthy controls [28]. In the current study, we did not observe any significant difference in any of the cT_FH_ cell subsets between the PAD group and the HC except that cT_FH2_ cells were significantly decreased following two doses of the vaccine, which would suggest that T_FH_ cell responses to SARS-CoV-2 infections are different from those to the SARC-CoV-2 vaccine.

In contrast, Sauerwein et al. [39] reported a significantly decreased percentage of antigen-specific cT_FH_ cells in CVID patients who made an antibody response to the vaccine and observed a defective function of cT_FH_ cells following activation with the SARS-CoV-2 peptide; titers of spike protein-specific IgG three times the detection limit or more were associated with intact vaccine-specific activation of CXCR5-negative CD4+ memory T cells, despite defective activation of circulating T follicular helper cells (cT_FH_). In contrast, CVID IgG non-responders showed defective vaccine-specific and superantigen-induced activation of both CD4+ T cell subsets. The reason for the discrepancy between our results and the other investigator is unclear. This could be due to the heterogeneity of CVID and having 15% of cases of other PADs in our group. Furthermore, these investigators did not examine other subpopulations of T_FH_ cells. The difference between these studies and ours is that we did not analyze SARS-CoV-2-specific T_FH_ cells. These observations may suggest an antigen-specific T_FH_ cell defect (lacunar deficiency). They reported that the induction of antigen-specific CD4^+^CD154^+^CD137^+^CXCR5^+^ peripheral T_FH_ (pT_FH_) cells by the COVID-19 vaccine was higher in CVID sero-responders than in sero-nonresponders. Further levels of pT_FH_ did not correlate with antibody response or avidity.

Fewer studies have reported T cell responses as compared to antibody responses following SARS-CoV-2 infection or following vaccination in PADs. In this study, we examined phenotypically identified various subsets of CD4 and CD8 T cells, SARS-CoV-2-specific T cells, and activation and IFN-γ production by T cell subsets. Our data show that SARS-CoV-2 tetramer-positive CD8 T_CM_ cells following two doses of the vaccine were significantly lower (*p* < 0.02) in the PAD patients as compared to the HC. Furthermore, following activation with SARS-CoV-2 spike protein, a significant increase in CD4+CD69+ (*p* < 0.01) and CD8+CD137 (*p* = 0.001) was observed in the PAD group as compared with the HC. However, CD4+CD137+IFN-γ+ cells were significantly decreased in the PAD group as compared to the HC. Therefore, following two doses of the SARS-CoV2 vaccine, though activation of CD4 and CD8 T cells is increased, a subset of CD4+ T cells is impaired in IFN-γ production.

Gernez et al. [34] reported normal IFN-γ production by T cells following stimulation with SARS-CoV-2 in patients with humoral immunodeficiency who received two doses of the SARS-CoV-2 vaccine. Steiner et al. [77] observed that all CVID sero-responders and 83% of non-seroresponders had a detectable polyfunctional T cell response. Pham et al. [37] evaluated 33 patients with humoral defects who received two doses of the vaccine and observed that IFN-γ production was positive in 24 (77.4%) of 31 patients. One of the earliest studies that prospectively evaluated the cellular immune response to the SARS-CoV-2 vaccine studied S-specific IFNγ T cell response by FluoroSpot in 18 patients with CVID before and after the 2nd dose of the SARS-CoV-2 vaccines. The cellular response rate was lower in CVID compared with the healthy controls, both after the first dose (50% vs. 88%) and after the second dose (83% vs. 98%).

Van Leeuwen et al. [60], in a large, prospective, controlled, multicenter study, reported the humoral and cellular immune responses after two doses of the mRNA-1273 COVID-19 vaccine in 505 patients with IEI and 192 controls. SARS-CoV-2-specific T-cell responses using the IFN-γ release assay were significantly lower in the CVID cohort than in the controls (67% vs. 88%). Milota et al. [56] assessed the T cell response to the vaccine in 21 patients with CVID following the vaccination series. One month after the second dose of the vaccine, CD4+ T cells in 46% of patients with CVID and 73% of individuals in the HC group responded to the S-RBD antigen in a short ex vivo stimulation and cytokine-production assays. Six months after the second dose of the vaccination, six of twelve patients with CVID and nine of fifteen HC had a CD4 T cell response. However, impaired T cell functions may be due to the use of freeze–thaw cells in CVID.

In-depth analyses of subsets of CD4 and CD8 T cells in PAD patients following the SARS-CoV-2 vaccine have not been published. Naïve CD4 and CD8 T cells, upon activation by an antigen, undergo activation, proliferation, and generation of effector and memory T cells [41,42,43]. Memory T cells that migrate to the lymph nodes are termed central memory (T_CM_), and those that migrate to extra-lymphoid tissue are termed effector memory (T_EM_). T effector memory cells that re-acquire CD45 are T_EMRA_. These subsets are distinct in their phenotype and functions [44].

We reported a detailed T cell subset analysis in a single CVID patient with mild clinical manifestations of SARS-CoV-2 infection who did not make SARS-CoV-2 antibodies [28]. CD4 T cells and CD4 naïve T cells increased; however, no changes were observed in CD4T_CM_ and CD4T_EMRA_. Furthermore, CD8T_EM_ decreased, and CD8T_EMRA_ increased as compared to the HC. In the current study, following the 2nd dose of the SARS-CoV-2 vaccine, CD4 naïve T cells were significantly decreased (*p* < 0.03), and CD8 T_CM_ were significantly increased (*p* < 0.01) in the PAD patients.

Patients with PADs, especially CVID, which is our major cohort of PADs, develop autoimmunity and autoimmune diseases [78]. Immunological tolerance, especially peripheral tolerance, is regulated by various members of the immunoregulatory lymphocyte club, including CD4Treg, CD8Treg, T_FR_, and Breg [54]. These regulatory lymphocytes have not been reported in PAD patients following SARS-CoV-2 infection except in a single CVID patient [28] or following SARS-CoV-2 vaccination in PADs. We reported normal CD4Treg, CD8Treg, and T_FR_ and increased Breg in a single patient with CVID following SARS-CoV-2 infection. Similarly, in the current study, we observed significantly increased Breg in the PAD group following the 2nd dose of the SARS-CoV-2 vaccine, whereas CD4Treg, CD8Treg, and T_FR_ were comparable between the HC and PAD patients.

Finally, we studied changes longitudinally in various subsets of CD4, CD8, T_FH_, B cells, regulatory lymphocytes, and SARS-CoV-2-specific T cells prior to and following the 1st and 2nd doses of the SARS-CoV-2 vaccine in two HC and three patients with CVID. CD19+ B cells, naïve B cells, and transitional B cells markedly increased following the 1st dose of the vaccine in the PAD patients but mostly recovered following the 2nd dose of the vaccine. In the HC, B cells and naïve B cells increased following the 2nd dose of the vaccine. The data on these two HC have been previously published [17]. In contrast, MZ B cells, switched memory B cells, CD21^low^, and plasmablasts increased following the 1st dose of the vaccine and returned towards the pre-vaccine level following the 2nd dose in the PAD patients. Amodio et al. [31] evaluated the immune response before and after the first and second doses (1 week after the second dose administration) of the BNT162b2 mRNA COVID-19 vaccine in both IEI and healthy controls. They reported that patients with IEI were able to develop a specific anti-spike antibody response following vaccination, although at a statistically significant lower magnitude compared to the healthy control. The data on T_FH_ subsets were variable in the CVID patients, except the proportions of T_FH1_ cells increased after the 2nd dose. The data on regulatory lymphocytes were interesting in that the level of CD4Treg cells in CVID following the 2nd dose were similar to the pre-vaccination level, whereas in the HC, they were markedly increased. T_FR_ cells increased following both the 1st and 2nd doses of vaccine and were comparable. In CVID, CD8Treg decreased after the 1st dose and, in two of the three CVID patients, decreased further. We have reported that CD8Treg suppresses the differentiation of T_FH_ cells and, therefore, might be responsible for the increased GC and plasmablasts observed following the 2nd dose of the vaccine. The data on subsets of CD4 and CD8 suggest relatively well-preserved T cells in our cohort of PAD.

The limitations of our study include a very small number of HC and CVID patients in the longitudinal study and, in the cohort group, a lack of data prior to vaccination with SARS-CoV-2. Unfortunately, when this study was started, the majority of HC and PAD patients had already received the SARS-CoV-2 vaccine. Furthermore, similar limitations have been observed in the majority of published studies of SARS-CoV-2 vaccine response in IEI. We also did not measure SARS-CoV-2 antibodies to correlate with the changes in various B cell subsets. Almost all of our patients were receiving IgRT, and Ig preparations contained various concentrations of anti-spike antibodies that varies among various manufacturers. It is unclear if those antibodies in the Ig preparation influence immune response.

In summary, the majority of published reports of more than 1000 patients with primary antibody deficiency (including CVID patients) demonstrate that approximately 65–80% of patients develop positive SARS-CoV-2 spike antibodies after two doses of the SARS-CoV-2 vaccine [32,60,78,79,80,81], and this rate further increased to 78% to almost 100% after the third vaccine dose [34,57,82]. An increase in GC B cells and plasmablasts in our PAD patients would also suggest that a large proportion of patients with PAD do mount a good antibody response to the SARS-CoV-2 vaccine. T cell subsets are relatively well-preserved, although the impairment of SARS-CoV-2-specific T cells has been reported in a small subset of CVID patients. Our study suggests that the majority of patients with PAD with an impaired response to the lipopolysaccharide vaccine (T-independent) do respond to the SARS-CoV-2 mRNA vaccines. Therefore, based upon our data and other published reports, we suggest that patients with PADs may receive two and, perhaps, regular vaccination with the SARS-CoV-2 vaccines as recommended by the CDC. The variability in response could be due to the heterogeneity of PADs (even monogenic IEI display different phenotypes) and suggests the need for individualized vaccination strategies and further investigation into the mechanisms in altered immune responses in PADs and other IEI.

## Figures and Tables

**Table 1:** Demographic data of patients with primary antibody deficiencies and healthy controls (HC).

Diagnosis	Age Range (Years) (Mean)	Gender	mRNA Vaccine
CVID (n = 30)	20–80 (62)	18 F, 12M	24 Pfizer, 6 Moderna
Hypogam (n = 8)	17–75 (56)	3F, 5M	6 Pfizer, 2 Moderna
sIgMD (n = 1)	56	M	Pfizer
XLA (n = 1)	74	M	Pfizer
IgG subclass (n = 1)	47	M	Moderna
SAD (n = 1)	69	F	Pfizer
Healthy Controls (n = 54)	23–72 (43)	38F, 16M	40 Pfizer, 14 Moderna

CVID = common variable immune deficiency; sIgMD = selective IgM deficiency; Hypogam = hypogammaglobulinemia; XLA = X-linked agammaglobulinemia; SAD = specific antibody deficiency.

## Data Availability

The data are stored in the Research laboratory locked cabinet and are available if need. They are kept for the next 7 years.

## References

[1] Baden L.R., El Sahly H.M., Essink B., Kotloff K., Frey S., Novak R., Diemert D., Spector S.A., Rouphael N., Creech C.B. (2021). Efficacy and Safety of the mRNA-1273 SARS-CoV-2 Vaccine. N. Engl. J. Med..

[2] Anderson E.J., Rouphael N.G., Widge A.T., Jackson L.A., Roberts P.C., Makhene M., Chappell J.D., Denison M.R., Stevens L.J., Pruijssers A.J. (2020). Safety and Immunogenicity of SARS-CoV-2 mRNA-1273 Vaccine in Older Adults. N. Engl. J. Med..

[3] McMenamin M.E., Nealon J., Lin Y., Wong J.Y., Cheung J.K., Lau E.H.Y., Wu P., Leung G.M., Cowling B.J. (2022). Vaccine effectiveness of one, two, and three doses of BNT162b2 and CoronaVac against COVID-19 in Hong Kong: A population-based observational study. Lancet Infect. Dis..

[4] Sette A., Crotty S. (2021). Adaptive immunity to SARS-CoV-2 and COVID-19. Cell.

[5] Tarke A., Sidney J., Kidd C.K., Dan J.M., Ramirez S.I., Yu E.D., Mateus J., da Silva Antunes R., Moore E., Rubiro P. (2021). Comprehensive analysis of T cell immunodominance and immunoprevalence of SARS-CoV-2 epitopes in COVID-19 cases. Cell Rep. Med..

[6] Zhang Z., Mateus J., Coelho C.H., Dan J.M., Moderbacher C.R., Galvez R.I., Cortes F.H., Grifoni A., Tarke A., Chang J. (2022). Humoral and cellular immune memory to four COVID-19 vaccines. Cell.

[7] Roltgen K., Boyd S.D. (2024). Antibody and B Cell Responses to SARS-CoV-2 Infection and Vaccination: The End of the Beginning. Annu. Rev. Pathol..

[8] Long Q.X., Liu B.Z., Deng H.J., Wu G.C., Deng K., Chen Y.K., Liao P., Qiu J.F., Lin Y., Cai X.F. (2020). Antibody responses to SARS-CoV-2 in patients with COVID-19. Nat. Med..

[9] Ni L., Ye F., Cheng M.L., Feng Y., Deng Y.Q., Zhao H., Wei P., Ge J., Gou M., Li X. (2020). Detection of SARS-CoV-2-Specific Humoral and Cellular Immunity in COVID-19 Convalescent Individuals. Immunity.

[10] Siracusano G., Pastori C., Lopalco L. (2020). Humoral Immune Responses in COVID-19 Patients: A Window on the State of the Art. Front. Immunol..

[11] Wajnberg A., Amanat F., Firpo A., Altman D.R., Bailey M.J., Mansour M., McMahon M., Meade P., Mendu D.R., Muellers K. (2020). Robust neutralizing antibodies to SARS-CoV-2 infection persist for months. Science.

[12] Woodruff M.C., Ramonell R.P., Nguyen D.C., Cashman K.S., Saini A.S., Haddad N.S., Ley A.M., Kyu S., Howell J.C., Ozturk T. (2020). Extrafollicular B cell responses correlate with neutralizing antibodies and morbidity in COVID-19. Nat. Immunol..

[13] Zhao J., Yuan Q., Wang H., Liu W., Liao X., Su Y., Wang X., Yuan J., Li T., Li J. (2020). Antibody Responses to SARS-CoV-2 in Patients With Novel Coronavirus Disease 2019. Clin. Infect. Dis..

[14] Chvatal-Medina M., Mendez-Cortina Y., Patino P.J., Velilla P.A., Rugeles M.T. (2021). Antibody Responses in COVID-19: A Review. Front. Immunol..

[15] Chen Z., John Wherry E. (2020). T cell responses in patients with COVID-19. Nat. Rev. Immunol..

[16] Sahin U., Muik A., Derhovanessian E., Vogler I., Kranz L.M., Vormehr M., Baum A., Pascal K., Quandt J., Maurus D. (2020). COVID-19 vaccine BNT162b1 elicits human antibody and T(H)1 T cell responses. Nature.

[17] Gupta S., Su H., Agrawal S. (2022). Immune Response to SARS-CoV-2 Vaccine in 2 Men. Int. Arch. Allergy Immunol..

[18] Ameratunga R., Longhurst H., Steele R., Lehnert K., Leung E., Brooks A.E.S., Woon S.T. (2021). Common Variable Immunodeficiency Disorders, T-Cell Responses to SARS-CoV-2 Vaccines, and the Risk of Chronic COVID-19. J. Allergy Clin. Immunol. Pract..

[19] Castano-Jaramillo L.M., Yamazaki-Nakashimada M.A., O’Farrill-Romanillos P.M., Muzquiz Zermeno D., Scheffler Mendoza S.C., Venegas Montoya E., Garcia Campos J.A., Sanchez-Sanchez L.M., Gamez Gonzalez L.B., Ramirez Lopez J.M. (2021). COVID-19 in the Context of Inborn Errors of Immunity: A Case Series of 31 Patients from Mexico. J. Clin. Immunol..

[20] Esenboga S., Ocak M., Akarsu A., Bildik H.N., Cagdas D., Iskit A.T., Tezcan I. (2021). COVID-19 in Patients with Primary Immunodeficiency. J. Clin. Immunol..

[21] Ho H.E., Mathew S., Peluso M.J., Cunningham-Rundles C. (2021). Clinical outcomes and features of COVID-19 in patients with primary immunodeficiencies in New York City. J. Allergy Clin. Immunol. Pract..

[22] Marcus N., Frizinsky S., Hagin D., Ovadia A., Hanna S., Farkash M., Maoz-Segal R., Agmon-Levin N., Broides A., Nahum A. (2020). Minor Clinical Impact of COVID-19 Pandemic on Patients with Primary Immunodeficiency in Israel. Front. Immunol..

[23] Meyts I., Bucciol G., Quinti I., Neven B., Fischer A., Seoane E., Lopez-Granados E., Gianelli C., Robles-Marhuenda A., Jeandel P.Y. (2021). Coronavirus disease 2019 in patients with inborn errors of immunity: An international study. J. Allergy Clin. Immunol..

[24] Milito C., Lougaris V., Giardino G., Punziano A., Vultaggio A., Carrabba M., Cinetto F., Scarpa R., Delle Piane R.M., Baselli L. (2021). Clinical outcome, incidence, and SARS-CoV-2 infection-fatality rates in Italian patients with inborn errors of immunity. J. Allergy Clin. Immunol. Pract..

[25] Quinti I., Lougaris V., Milito C., Cinetto F., Pecoraro A., Mezzaroma I., Mastroianni C.M., Turriziani O., Bondioni M.P., Filippini M. (2020). A possible role for B cells in COVID-19? Lesson from patients with agammaglobulinemia. J. Allergy Clin. Immunol..

[26] Shields A.M., Burns S.O., Savic S., Richter A.G., UK PIN COVID-19 Consortium (2021). COVID-19 in patients with primary and secondary immunodeficiency: The United Kingdom experience. J. Allergy Clin. Immunol..

[27] Soresina A., Moratto D., Chiarini M., Paolillo C., Baresi G., Foca E., Bezzi M., Baronio B., Giacomelli M., Badolato R. (2020). Two X-linked agammaglobulinemia patients develop pneumonia as COVID-19 manifestation but recover. Pediatr. Allergy Immunol..

[28] Gupta S., Su H., Narsai T., Agrawal S. (2021). SARS-CoV-2-Associated T-Cell Responses in the Presence of Humoral Immunodeficiency. Int. Arch. Allergy Immunol..

[29] Gupta S., Agrawal S., Sandoval A., Su H., Tran M., Demirdag Y. (2022). SARS-CoV-2-Specific and Functional Cytotoxic CD8 Cells in Primary Antibody Deficiency: Natural Infection and Response to Vaccine. J. Clin. Immunol..

[30] Pulvirenti F., Fernandez Salinas A., Milito C., Terreri S., Piano Mortari E., Quintarelli C., Di Cecca S., Lagnese G., Punziano A., Guercio M. (2021). B Cell Response Induced by SARS-CoV-2 Infection Is Boosted by the BNT162b2 Vaccine in Primary Antibody Deficiencies. Cells.

[31] Amodio D., Ruggiero A., Sgrulletti M., Pighi C., Cotugno N., Medri C., Morrocchi E., Colagrossi L., Russo C., Zaffina S. (2021). Humoral and Cellular Response following Vaccination with the BNT162b2 mRNA COVID-19 Vaccine in Patients Affected by Primary Immunodeficiencies. Front. Immunol..

[32] Arroyo-Sanchez D., Cabrera-Marante O., Laguna-Goya R., Almendro-Vazquez P., Carretero O., Gil-Etayo F.J., Suarez-Fernandez P., Perez-Romero P., Rodriguez de Frias E., Serrano A. (2022). Immunogenicity of Anti-SARS-CoV-2 Vaccines in Common Variable Immunodeficiency. J. Clin. Immunol..

[33] Fernandez Salinas A., Piano Mortari E., Terreri S., Milito C., Zaffina S., Perno C.F., Locatelli F., Quinti I., Carsetti R. (2022). Impaired memory B-cell response to the Pfizer-BioNTech COVID-19 vaccine in patients with common variable immunodeficiency. J. Allergy Clin. Immunol..

[34] Gernez Y., Murugesan K., Cortales C.R., Banaei N., Hoyte L., Pinsky B.A., Lewis D.B., Pham M.N. (2022). Immunogenicity of a third COVID-19 messenger RNA vaccine dose in primary immunodeficiency disorder patients with functional B-cell defects. J. Allergy Clin. Immunol. Pract..

[35] Hagin D., Freund T., Navon M., Halperin T., Adir D., Marom R., Levi I., Benor S., Alcalay Y., Freund N.T. (2021). Immunogenicity of Pfizer-BioNTech COVID-19 vaccine in patients with inborn errors of immunity. J. Allergy Clin. Immunol..

[36] Murray C.E., O’Brien C., Alamin S., Phelan S.H., Argue R., Kiersey R., Gardiner M., Naughton A., Keogh E., Holmes P. (2023). Cellular and humoral immunogenicity of the COVID-19 vaccine and COVID-19 disease severity in individuals with immunodeficiency. Front. Immunol..

[37] Pham M.N., Murugesan K., Banaei N., Pinsky B.A., Tang M., Hoyte E., Lewis D.B., Gernez Y. (2022). Immunogenicity and tolerability of COVID-19 messenger RNA vaccines in primary immunodeficiency patients with functional B-cell defects. J. Allergy Clin. Immunol..

[38] Sauerwein K.M.T., Geier C.B., Stemberger R.F., Akyaman H., Illes P., Fischer M.B., Eibl M.M., Walter J.E., Wolf H.M. (2022). Antigen-Specific CD4(+) T-Cell Activation in Primary Antibody Deficiency after BNT162b2 mRNA COVID-19 Vaccination. Front. Immunol..

[39] Sauerwein K.M.T., Geier C.B., Stemberger R.F., Rossmanith R., Akyaman H., Illes P., Fischer M.B., Eibl M.M., Walter J.E., Wolf H.M. (2023). Functionally impaired antibody response to BNT162b2 booster vaccination in CVID IgG responders. J. Allergy Clin. Immunol..

[40] Conley M.E., Notarangelo L.D., Etzioni A. (1999). Diagnostic criteria for primary immunodeficiencies. Representing PAGID (Pan-American Group for Immunodeficiency) and ESID (European Society for Immunodeficiencies). Clin. Immunol..

[41] Sallusto F., Lenig D., Forster R., Lipp M., Lanzavecchia A. (1999). Two subsets of memory T lymphocytes with distinct homing potentials and effector functions. Nature.

[42] Weninger W., Crowley M.A., Manjunath N., von Andrian U.H. (2001). Migratory properties of naive, effector, and memory CD8(+) T cells. J. Exp. Med..

[43] van Lier R.A., ten Berge I.J., Gamadia L.E. (2003). Human CD8(+) T-cell differentiation in response to viruses. Nat. Rev. Immunol..

[44] Gupta S. (2005). Molecular mechanisms of apoptosis in the cells of the immune system in human aging. Immunol. Rev..

[45] Ueno H. (2016). Human Circulating T Follicular Helper Cell Subsets in Health and Disease. J. Clin. Immunol..

[46] Victora G.D., Schwickert T.A., Fooksman D.R., Kamphorst A.O., Meyer-Hermann M., Dustin M.L., Nussenzweig M.C. (2010). Germinal center dynamics revealed by multiphoton microscopy with a photoactivatable fluorescent reporter. Cell.

[47] Crotty S. (2019). T Follicular Helper Cell Biology: A Decade of Discovery and Diseases. Immunity.

[48] Morita R., Schmitt N., Bentebibel S.E., Ranganathan R., Bourdery L., Zurawski G., Foucat E., Dullaers M., Oh S., Sabzghabaei N. (2011). Human blood CXCR5(+)CD4(+) T cells are counterparts of T follicular cells and contain specific subsets that differentially support antibody secretion. Immunity.

[49] LeBien T.W., Tedder T.F. (2008). B lymphocytes: How they develop and function. Blood.

[50] Kurosaki T. (2010). B-lymphocyte biology. Immunol. Rev..

[51] Pieper K., Grimbacher B., Eibel H. (2013). B-cell biology and development. J. Allergy Clin. Immunol..

[52] Martin F., Kearney J.F. (2002). Marginal-zone B cells. Nat. Rev. Immunol..

[53] Inoue T., Kurosaki T. (2024). Memory B cells. Nat. Rev. Immunol..

[54] Gupta S., Demirdag Y., Gupta A.A. (2022). Members of the Regulatory Lymphocyte Club in Common Variable Immunodeficiency. Front. Immunol..

[55] Padron G.T., Hernandez-Trujillo V.P. (2023). Autoimmunity in Primary Immunodeficiencies (PID). Clin. Rev. Allergy Immunol..

[56] Milota T., Smetanova J., Skotnicova A., Rataj M., Lastovicka J., Zelena H., Parackova Z., Fejtkova M., Kanderova V., Fronkova E. (2023). Clinical Outcomes, Immunogenicity, and Safety of BNT162b2 Vaccine in Primary Antibody Deficiency. J. Allergy Clin. Immunol. Pract..

[57] Ainsua-Enrich E., Pedreno-Lopez N., Bracke C., Avila-Nieto C., Rodriguez de la Concepcion M.L., Pradenas E., Trinite B., Marfil S., Miranda C., Gonzalez S. (2022). Kinetics of immune responses elicited after three mRNA COVID-19 vaccine doses in predominantly antibody-deficient individuals. iScience.

[58] Nielsen B.U., Drabe C.H., Barnkob M.B., Johansen I.S., Hansen A.K.K., Nilsson A.C., Rasmussen L.D. (2022). Antibody response following the third and fourth SARS-CoV-2 vaccine dose in individuals with common variable immunodeficiency. Front. Immunol..

[59] Antoli A., Rocamora-Blanch G., Framil M., Mas-Bosch V., Navarro S., Bermudez C., Martinez-Yelamos S., Dopico E., Calatayud L., Garcia-Munoz N. (2022). Evaluation of Humoral and Cellular Immune Responses to the SARS-CoV-2 Vaccine in Patients With Common Variable Immunodeficiency Phenotype and Patient Receiving B-Cell Depletion Therapy. Front. Immunol..

[60] van Leeuwen L.P.M., GeurtsvanKessel C.H., Ellerbroek P.M., de Bree G.J., Potjewijd J., Rutgers A., Jolink H., van de Veerdonk F., van Gorp E.C.M., de Wilt F. (2022). Immunogenicity of the mRNA-1273 COVID-19 vaccine in adult patients with inborn errors of immunity. J. Allergy Clin. Immunol..

[61] Thorarinsdottir K., Camponeschi A., Cavallini N., Grimsholm O., Jacobsson L., Gjertsson I., Martensson I.L. (2016). CD21(-/low) B cells in human blood are memory cells. Clin. Exp. Immunol..

[62] Keller B., Strohmeier V., Harder I., Unger S., Payne K.J., Andrieux G., Boerries M., Felixberger P.T., Landry J.J.M., Nieters A. (2021). The expansion of human T-bet(high)CD21(low) B cells is T cell dependent. Sci. Immunol..

[63] Yang R., Avery D.T., Jackson K.J.L., Ogishi M., Benhsaien I., Du L., Ye X., Han J., Rosain J., Peel J.N. (2022). Human T-bet governs the generation of a distinct subset of CD11c(high)CD21(low) B cells. Sci. Immunol..

[64] Gjertsson I., McGrath S., Grimstad K., Jonsson C.A., Camponeschi A., Thorarinsdottir K., Martensson I.L. (2022). A close-up on the expanding landscape of CD21-/low B cells in humans. Clin. Exp. Immunol..

[65] Pape K.A., Dileepan T., Kabage A.J., Kozysa D., Batres R., Evert C., Matson M., Lopez S., Krueger P.D., Graiziger C. (2021). High-affinity memory B cells induced by SARS-CoV-2 infection produce more plasmablasts and atypical memory B cells than those primed by mRNA vaccines. Cell Rep..

[66] Arumugakani G., Wood P.M., Carter C.R. (2010). Frequency of Treg cells is reduced in CVID patients with autoimmunity and splenomegaly and is associated with expanded CD21lo B lymphocytes. J. Clin. Immunol..

[67] Rakhmanov M., Keller B., Gutenberger S., Foerster C., Hoenig M., Driessen G., van der Burg M., van Dongen J.J., Wiech E., Visentini M. (2009). Circulating CD21low B cells in common variable immunodeficiency resemble tissue homing, innate-like B cells. Proc. Natl. Acad. Sci. USA.

[68] Breitfeld D., Ohl L., Kremmer E., Ellwart J., Sallusto F., Lipp M., Forster R. (2000). Follicular B helper T cells express CXC chemokine receptor 5, localize to B cell follicles, and support immunoglobulin production. J. Exp. Med..

[69] Kim C.H., Rott L.S., Clark-Lewis I., Campbell D.J., Wu L., Butcher E.C. (2001). Subspecialization of CXCR5+ T cells: B helper activity is focused in a germinal center-localized subset of CXCR5+ T cells. J. Exp. Med..

[70] Nurieva R.I., Chung Y., Martinez G.J., Yang X.O., Tanaka S., Matskevitch T.D., Wang Y.H., Dong C. (2009). Bcl6 mediates the development of T follicular helper cells. Science.

[71] Chung Y., Tanaka S., Chu F., Nurieva R.I., Martinez G.J., Rawal S., Wang Y.H., Lim H., Reynolds J.M., Zhou X.H. (2011). Follicular regulatory T cells expressing Foxp3 and Bcl-6 suppress germinal center reactions. Nat. Med..

[72] Wollenberg I., Agua-Doce A., Hernandez A., Almeida C., Oliveira V.G., Faro J., Graca L. (2011). Regulation of the germinal center reaction by Foxp3+ follicular regulatory T cells. J. Immunol..

[73] Ma C.S., Wong N., Rao G., Avery D.T., Torpy J., Hambridge T., Bustamante J., Okada S., Stoddard J.L., Deenick E.K. (2015). Monogenic mutations differentially affect the quantity and quality of T follicular helper cells in patients with human primary immunodeficiencies. J. Allergy Clin. Immunol..

[74] Bentebibel S.E., Lopez S., Obermoser G., Schmitt N., Mueller C., Harrod C., Flano E., Mejias A., Albrecht R.A., Blankenship D. (2013). Induction of ICOS+CXCR3+CXCR5+ TH cells correlates with antibody responses to influenza vaccination. Sci. Transl. Med..

[75] Locci M., Havenar-Daughton C., Landais E., Wu J., Kroenke M.A., Arlehamn C.L., Su L.F., Cubas R., Davis M.M., Sette A. (2013). Human circulating PD-1+CXCR3-CXCR5+ memory Tfh cells are highly functional and correlate with broadly neutralizing HIV antibody responses. Immunity.

[76] Kasahara T.M., Gupta S. (2022). CD8 Treg-Mediated Suppression of Naive CD4+ T Cell Differentiation into Follicular Helper T Cells. Int. Arch. Allergy Immunol..

[77] Steiner S., Schwarz T., Corman V.M., Jeworowski L.M., Bauer S., Drosten C., Scheibenbogen C., Hanitsch L.G. (2023). Impaired B Cell Recall Memory and Reduced Antibody Avidity but Robust T Cell Response in CVID Patients after COVID-19 Vaccination. J. Clin. Immunol..

[78] Squire J., Joshi A. (2021). Seroconversion after coronavirus disease 2019 vaccination in patients with immune deficiency. Ann. Allergy Asthma Immunol..

[79] Durkee-Shock J.R., Keller M.D. (2022). Immunizing the imperfect immune system: Coronavirus disease 2019 vaccination in patients with inborn errors of immunity. Ann. Allergy Asthma Immunol..

[80] Goda V., Krivan G., Kulcsar A., Gonczi M., Tasnady S., Matula Z., Nagy G., Beko G., Horvath M., Uher F. (2022). Specific Antibody and the T-Cell Response Elicited by BNT162b2 Boosting after Two ChAdOx1 nCoV-19 in Common Variable Immunodeficiency. Front. Immunol..

[81] Pulvirenti F., Di Cecca S., Sinibaldi M., Piano Mortari E., Terreri S., Albano C., Guercio M., Sculco E., Milito C., Ferrari S. (2022). T-Cell Defects Associated to Lack of Spike-Specific Antibodies after BNT162b2 Full Immunization Followed by a Booster Dose in Patients with Common Variable Immune Deficiencies. Cells.

[82] Shields A.M., Faustini S.E., Hill H.J., Al-Taei S., Tanner C., Ashford F., Workman S., Moreira F., Verma N., Wagg H. (2022). Increased Seroprevalence and Improved Antibody Responses following Third Primary SARS-CoV-2 Immunisation: An Update from the COV-AD Study. Front. Immunol..

